# Use of the intraosseous screw for unilateral upper molar distalization and found well balanced occlusion

**DOI:** 10.1186/1746-160X-2-38

**Published:** 2006-11-09

**Authors:** Ibrahim Erhan Gelgor, Ali Ihya Karaman, Tamer Buyukyilmaz

**Affiliations:** 1 Kirikkale University, Faculty of Dentistry, Department of Orthodontics, Kirikkale, Turkey; 2 Selcuk University, Faculty of Dentistry, Department of Orthodontics, Konya, Turkey; 3 Cukurova University, Faculty of Dentistry, Department of Orthodontics, Adana, Turkey

## Abstract

**Background:**

The aim of this study was to present a temporary anchorage device with intraosseous screw for unilateral molar distalization to make a space for the impacted premolar and to found well balanced occlusion in a case.

**Case presentation:**

A 13-year-old male who have an impacted premolar is presented with skeletal Class I and dental Class 2 relationship. The screw was placed and immediately loaded to distalize the left upper first and second molar. The average distalization time to achieve an overcorrected Class I molar relationship was 3.6 months. There was no change in overjet, overbite, or mandibular plane angle measurements. Mild protrusion (0.5 mm) of the upper left central incisor was also recorded.

**Conclusion:**

Immediately loaded intraosseous screw-supported anchorage unit was successful in achieving sufficient unilateral molar distalization without anchorage loss. This treatment procedure was an alternative treatment to the extraction therapy.

## Background

In the treatment of Angle Class II malocclusions, with well-aligned lower teeth and a mandible in sagitally normal position, upper anterior crowding and excessive overjet can be treated with either distalization or extraction of upper posterior teeth. Newly developed orthodontic mechanics and their ease of application enabled widespread use of nonextraction therapies[1].

Conventional extraoral appliances are usually used for supporting maxillary molar anchorage or for distalization purposes. However, patient cooperation is a serious problem that has to be dealt with and moreover, orthodontic mechanics requiring minimal patient cooperation are desirable [2,3]. A number of treatment protocols that minimize the need for patient compliance have been suggested previously [4-12]. These techniques effectively distalize the maxillary molars, however, in most of these studies anchorage loss is unavoidable characterized by maxillary incisor protrusion, an increase in overjet, and decrease in overbite [6,7,11].

In recent years, studies have been directed toward the use of osseointegrated implants [3,12-14], onplants [15], and intraosseous screws [1] as anchorage units in orthodontic patients.

Use of intraosseous screws for temporary orthodontic anchorage devices is a new area of research [1,3,16]. Creekmore and Eklund [16] used a Vitallium screw for intrusion of the upper incisors. Park et al [17] successfully used maxillary microscrews for treatment of openbite malocclusion. Liou et al [18] and Park et al [19,20] carried out en masse distalization of upper and lower posterior teeth using microscrew implant anchorage. In our previous study [1], we prepared an anchorage unit for bilateral upper molar distalization by placing an intraosseous screw in twenty five cases. During the following 4.6 months, both the first and second molars were distalized into an overcorrected Class I relationship without major anchorage loss.

The aim of this study was to present use of the intraosseous screw for unilateral upper molar distalization in a case.

## Case presentation

**Figure 1:**
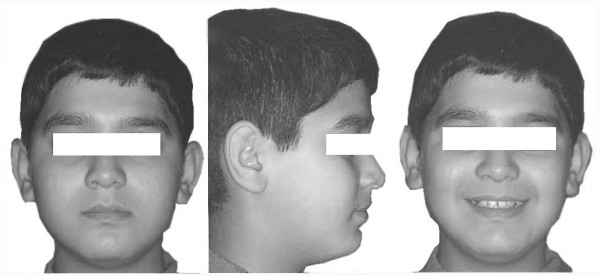
Pretreatment extraoral photographs of a 13-year-old male patient.

A 13-year-old male presented skeletal Class I relationship. The patient's profile was mild convex. Vertical facial proportions were normal, and there were no significant asymmetries (Figure 1).

**Figure 2:**
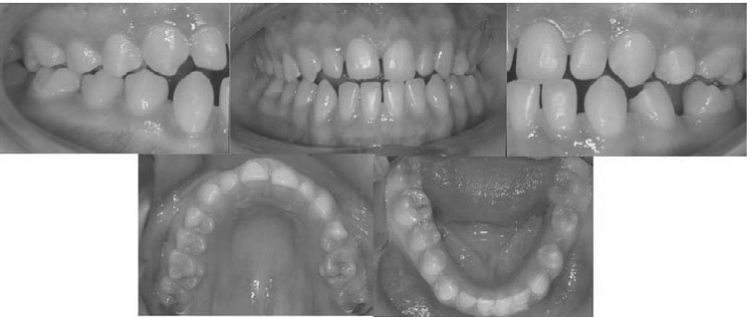
Pretreatment intraoral photographs of the patient.

**Figure 3:**
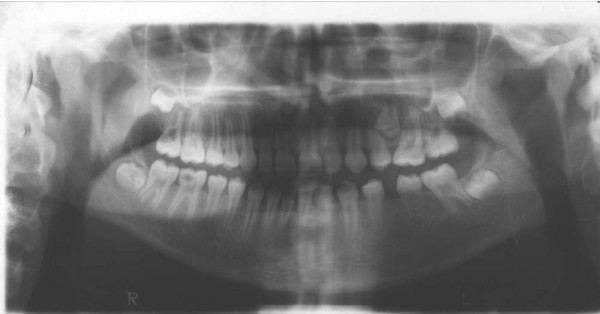
Pretreatment panoramic radiograph of the patient.

A full complement of permanent teeth was present except left lower first molar. There was a huge caries in the lower right first molar. Upper left second premolar was impacted. In centric occlusion canine relationships were Class I, and the incisors were in teeth a teeth relation. Both the maxillary and the mandibular arches exhibited moderate teeth disorderliness. Oral hygiene was moderate (Figures 2, and 3).

**Figure 4:**
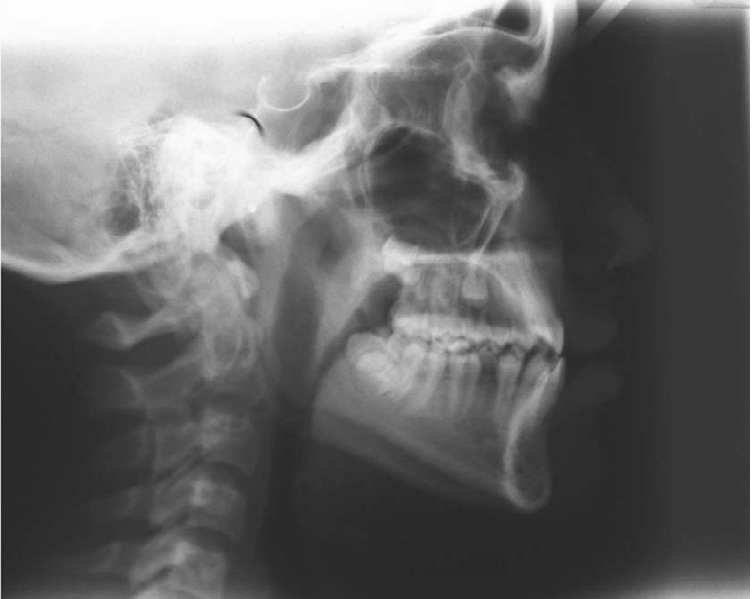
Pretreatment cephalometric radiograph of the patient.

In pretreatment cephalometric evaluation (Figure 4, Table 1); the maxilla was normal to the cranial base (SNA 86°), and in centric occlusion the mandible was normal position to the cranial base (SNB 84°). The ANB (2°) indicated a Class I skeletal relationship. The maxillary incisors were slightly upright, while the mandibular incisors were somewhat protrusive. The mandibular plane was normal relative to cranial base (SN-MP 31°).

**Table 1:** Cephalometric Analysis

	Pre Treatment	After Distalization	Post Treatment
**SKELETAL**			
SNA (deg)	86	86	86
SNB (deg)	84	84	84
ANB (deg)	2	2	2
SN-PP (deg)	5.2	5.2	5.2
SN-MP (deg)	31	31	31
N-ANS (mm)	50	50	50
ANS-Me (mm)	69	69	69
**DENTAL**			
U1-SN (deg)	100	101	104
U4-PP (deg)	80.3	83.1	81
U6-PP (deg)	75.4	81.2	75.8
L1-MP (deg)	94	94	90
U1-APo (mm)	1.5	2	2.5
L1-APo (mm)	3	3	-1
S ⊥ U6b	24.7	20.8	21.2
**SOFT TISSUE**			
ULip-APo (mm)	-3	-3	-3
LLip-APo (mm)	-2	-2	-2

### Treatment objectives

1. to establish Class I molar relationship.

2. to eliminate maxillary and mandibular arch disorders.

3. to erupt upper left second premolar because of the patient's rejection of surgically extraction of the impacted premolar.

4. to correct overbite, and overjet.

5. to align arches including midlines.

6. to constitute a good smile aesthetic.

The criteria for unilateral intraoral molar distalization were included;

• Skeletal Class I, unilateral Class II molar and canine relationship;

• Minimal or no crowding in the mandibular arch;

• Existence of bilateral 1^st ^or 2^nd ^premolar teeth;

• Rejection of surgically extraction of the impacted premolar;

• Rejection of headgear wear;

• Good oral hygiene.

### The intraosseous screw and insertion procedure

The intraosseous screw (IMF Stryker, Leibinger, Germany) is a pure titanium one-piece device with an endosseous body and intraoral neck section. In this study, 1.8 mm diameter and 14-mm length screws were used.

**Figure 5:**
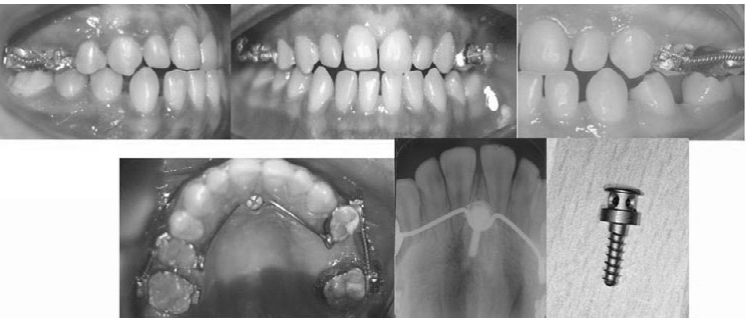
After distalization intraoral photographs and occlusal radiograph of the patient and intraosseous screw.

The intraosseous screw was placed behind the incisive canal at a safe distance from the midpalatal suture following the palatal anatomy. To facilitate this application under local anesthesia, a syringe was placed in the incisive canal for reference, and a 1.5-mm-diameter hole was drilled five mm behind the syringe and three mm to the right or left of the raphe. The procedure took 5–8 minutes and a mucoperiostal opening flap was not needed [1] (Figure 5).

### Fabrication of the distalization appliance

After healing, an impression was obtained with the screw in place, and a plaster model was prepared.

Upper right and left first premolar and first molar bands that had 0.018-inch brackets and 0.030-inch tubes were fitted to the teeth on the dental cast. A 0.036-inch (0.9 mm) stainless steel transpalatal arch (TPA) was prepared between the first premolars, with a "U" bend touching the screw. The TPA was soldered to the bands, the bands were cemented onto the premolars, and the U bend was bonded to the intraoral neck section of the screw using light-cured composite resin [1], then bilateral sectional arches (0.016 × 0.022-inch stainless steel) and 0.036-inch nickel-titanium open-coil springs were inserted between upper left first premolar and first molar with a continuous force of 250 g and at the right side passively (Figure 5).

**Figure 6:**
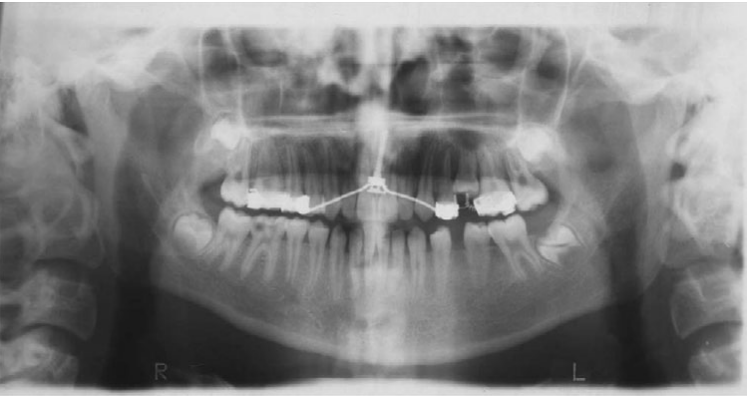
After distalization panoramic radiograph of the patient.

**Figure 7:**
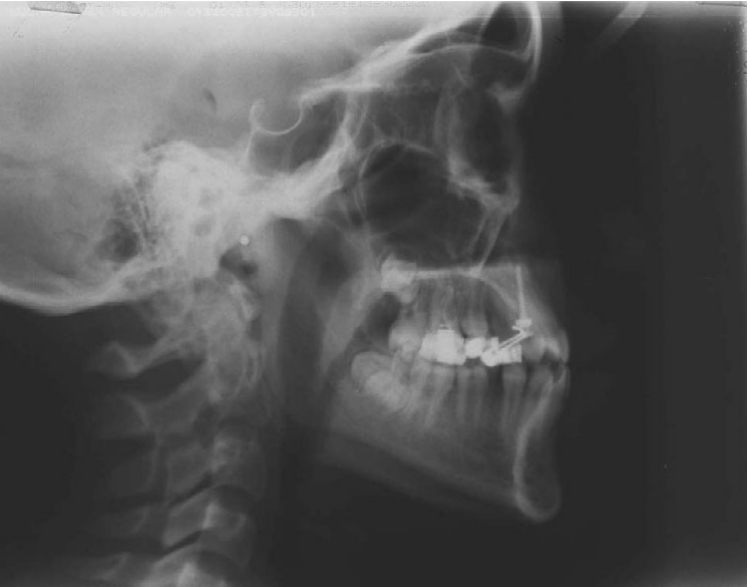
After distalization cephalometric radiograph of the patient.

The patient was seen every 4 weeks, and the force level of the coil spring was checked and activated when necessary. When upper left first molar was moved into an overcorrected Class I relationship by approximately 2 mm, the distalization was ended (Figures 5, 6, 7).

A simple radio opaque cap was applied to the left first molar when taking cephalogram to differentiating the left from the right on ceph.

After distalization, the following treatment was established:

Maxillary and mandibular fixed appliances (.018 × .025 inch slot) were used. After initial leveling and alignment with round arch wires in upper and lower dental arch, a .016 × .022 inch ss utility arch was used for protrusion of the upper incisors. For retrusion of the mandibular incisors .016 × .022 inch continue arch with lingual root torque in incisor region and Class III elastics were used. Fixed appliance treatment was completed in 14 months.

## Results

The first molar was successfully distalized into an over corrected Cl I relationship and the needed space for the upper left second premolar eruption was gained. Distalization time was 3.6 months (Figures 5, 6, 7). The insertion procedure of the screws was quick and simple. The patient reported no pain required analgesic after the insertion and during the distalization period. Depending on the level of around the screw hygiene, the adjacent tissues showed no inflammation.

The screw was stabile right after the insertion. After the distalization period, no screw mobility was recorded.

**Figure 8:**
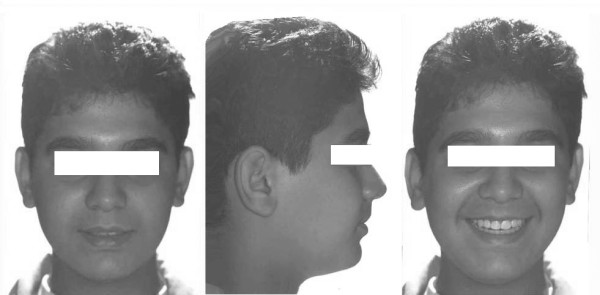
Posttreatment extraoral photographs of the patient.

**Figure 9:**
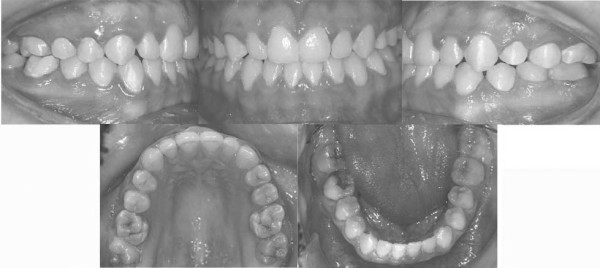
Posttreatment intraoral photographs of the patient.

**Figure 10:**
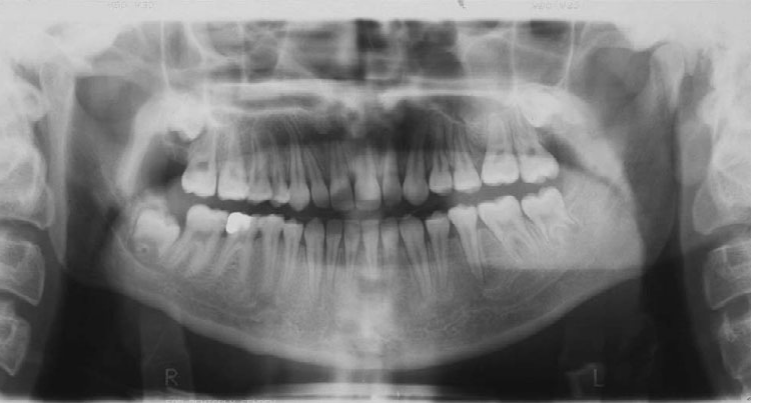
Posttreatment panoramic radiograph of the patient.

**Figure 11:**
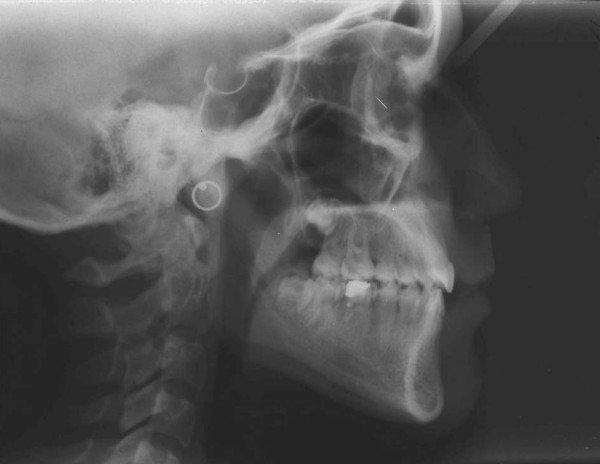
Posttreatment cephalometric radiograph of the patient.

**Figure 12:**
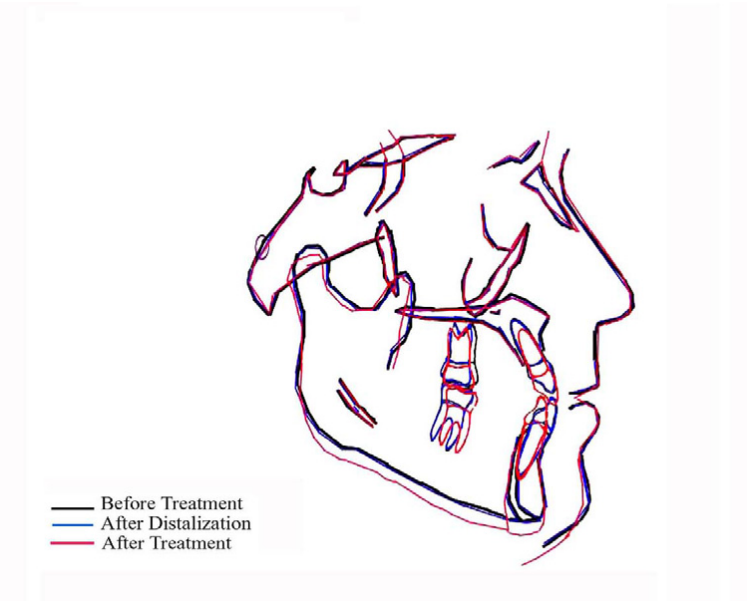
Cephalometric superimposition.

End of treatment, a positive overjet and overbite was established. Good torque control was maintained while the mandibular incisors were retracted resulting in better incisal inclination after treatment. Correction of the malocclusion was accomplished with dental movements (Figures 8, 9, 10, 11, 12). Caries in the lower right first molar was restored with the amalgam filling.

### Cephalometric analysis

After distalization, the maxillary left first molar distalization was 3.9 mm when measured at the mesial buccal cusp tip. The maxillary left molar crown tipped distally of 5.80°. In the same treatment phase, the upper left first premolar tipped mesially of 2.8°. Maxillary left incisor proclined approximately 1°. The incisor was advanced of 0.5 mm at incisal edge. Vertical and sagital dimensions remained virtually unchanged (Table 1, Figure 12).

## Discussion

Anchorage control is of great importance in orthodontic treatment. In the treatment of Angle Class II malocclusions, with Class I skeletal relationship, upper anterior crowding or excessive overjet can be treated with either unilateral/bilateral upper premolar extraction or distalization of upper posterior teeth consolidation of the anterior teeth [1]. The extractions create generally bad emotional effects on the patients that fear of dentist is present nearly in all people. Closing of the extraction spaces need extra time in all orthodontic treatment. The researchers have used intaroral distalization mechanics alternatively to the extraction treatment but anchorage loss has shown by the use of a lot of appliances with the significant maxillary incisor proclination and increased in overjet at the end of the distalization. [5,10,11].

In the present study, extraction of the impacted premolar will make simpler the all treatment. However, the patient didn't want to the extraction process. We decided using the intraosseous screw supported molar distalization appliance to regain the space for eruption of the impacted tooth. The patient and his parents were agreeing to this procedure cause of minimal risks of this treatment.

We used the intramaxillary fixation screw alternatively to the osseointegrated implants that would provide enough stability to actively distalize maxillary molars uni-or bilaterally, tolerate immediate loading, and provide anchorage in general. The desired immobility of this screw was relied on the grooves to establish mechanical locking between the screw and the surrounding bone. The insertion procedure took 5–8 minutes and no needed opening mucoperiostal flap. They weren't seen inflammation, bleeding or excessive pain in the adjacent tissues to the screw and the screw showed primary stability. These were advantages of the screw according to surgically extraction of upper left second premolar. The distalization system efficiently distalized the maxillary molar teeth to a Class I relationship. This distalization occurred without any cooperation problems for the patient. Thus the second premolar tooth erupted to occlusion free of problems.

In our study there was present slightly anchorage loss as defined by maxillary incisor proclination (1°) and increased in overjet that occurred at the end of movement (mean 0.5 mm), but these rates were unimportant clinically. However, we were again protruded of the upper incisors at fixed treatment stage to provide an ideal overbite, and overjet relationship.

## Conclusion

This study has shown the properties and action of an anchorage device with an intraosseous screw for unilateral upper molar distalization in a patient who has rejected surgically extraction his impacted premolar. The esthetic and compliance free nature of the distalization system seems to be superior to the alternative requirement of headgear and Class II elastics as maximum anchorage is required. In addition to the relative ease of placement and removal, other aspects of system also make this procedure more acceptable to the patients.

## Competing interests

The author(s) declare that they have no competing interests.

## Authors' contributions

IEG, AIK and TB performed the described operation and participated in the paper design.

IEG drafted the manuscript and wrote the text.

All authors read and approved the final manuscript.
